# The challenge of adults with phenylketonuria who have been lost to care; a single center's attempt to reach those diagnosed with PKU over 60 years of newborn screening

**DOI:** 10.1016/j.ymgmr.2024.101099

**Published:** 2024-06-08

**Authors:** S. Sacharow, E. Zhu, S. Hollander

**Affiliations:** a Boston Children's Hospital, Boston, MA 02115, United States of America; b Harvard Medical School, Boston, MA 02115, United States of America

**Keywords:** Phenylketonuria, PKU, Lost to care, Newborn screening, Lost-to-followup, Adults, Guidelines, Education

## Abstract

**Background:**

Those diagnosed with PKU in the early years of newborn screening (NBS) were often discharged from clinic in childhood. Long-term lost to clinic patients may be impacted by untreated PKU and uninformed about current recommendations. We aimed to contact adults away from clinic for 5–50+ years, share current recommendations, offer clinical care, and elicit factors underlying not returning to clinic.

**Methods:**

Former patients were identified and offered a virtual meeting with a physician and dietitian for structured interview and education about current guidelines and treatments.

**Results:**

We identified 53 eligible patients who had PKU and had not returned to clinic in ≥5 years. Of those 53, 27 were successfully contacted, 16 completed the educational intervention, and 5/16 returned to clinic. Reasons for having been away from clinic included discharge from clinic in childhood and inadequate insurance coverage. Experiences varied and some denied negative impacts after diet discontinuation. Individuals expressed a desire for convenient treatments that aligned with overall health goals. Most participants who completed the educational intervention expressed interest in returning to clinic; however, most did not return within the timeframe of the project. All 27 individuals successfully contacted agreed to be re-contacted with future updates or research opportunities.

**Discussion:**

We successfully contacted half of individuals identified as having been lost to clinic follow-up long-term. Limitations included inability to make initial contact, and unwillingness to re-engage by some we reached. Those who agreed to participation desired ongoing PKU clinic and community connection. This experience will inform our process to engage current patients and re-engage those currently lost to care.

## 1 Background

Phenylketonuria (PKU) [OMIM 261600] is a rare inherited disorder caused by phenylalanine hydroxylase (PAH) deficiency which results in accumulation of blood phenylalanine (Phe). PKU is estimated to occur in 1:10,000 to 1:15,000 people in the United States [1]. Untreated PKU in infancy and childhood may result in irreversible cognitive disability, and after the critical period for neurodevelopment can cause neuropsychiatric symptoms with a reversible component, including executive function deficits, anxiety and depression, as well as chronic neurological features like headaches and tremors [2].

PKU was first described in 1934. In the 1950s, a low Phe diet with Phe-reduced protein replacement formula became the standard of care for PKU. The Guthrie test, developed in 1961, allowed for monitoring of patients with PKU on dietary therapy and for the screening of newborns [3,4]. Massachusetts was the first state to carry out a pilot program to screen newborns for PKU in late 1962. Due to the early success in identifying babies with PKU pre-symptomatically, Massachusetts became the first state to mandate newborn screening (NBS) for PKU in 1963, with 32 states having NBS laws by 1965. By 1975, 90% of babies born in the U.S. were screened for PKU; however, it was not until 1985 that NBS for PKU was mandated in every state in the U.S. [5,6]

Since the initiation of NBS for PKU, management recommendations have significantly changed due to advancing knowledge about this disorder as well as new treatment modalities. During the early years of PKU diet therapy, the only option for formula was an amino acid mixture and low-protein specialty foods were extremely limited, leading to a diet that was less palatable and less varied than the present.

From 1962 until 1991, patients were often advised to only follow dietary treatment in early childhood until age six, at which point treatment could be safely stopped [7]. In 1991, the National PKU Collaborative Study reported that patients who discontinued diet at age six had lower cognitive scores on standardized measures compared to those who continued treatment past age six [8]. NIH guidelines now recommend treatment for life with the goal of Phe levels between 120 and 360 μmol/L [9].

Despite the recommendation for life-long treatment 24 years ago, the majority of adults with PKU in the U.S. are likely not on treatment. Berry et al. estimated that approximately 52% of individuals diagnosed with PKU at birth are not attending clinic, with this number jumping to 77% for patients between ages 25 to 45 years [10]. An online survey of PKU clinics in the U.S. estimated that 32% of patients were not currently actively managed by responding clinics, and this number increased to 55% for patients aged 30 years or older [11].

In 2017 there was a concerted effort to create best-practice recommendations by the PKU Lost to Follow-Up Recommendations Group, based on a survey of individuals with PKU and expert meetings with clinicians. They presented six practice recommendations for re-engaging patients with PKU: informing patients of current treatment guidelines, raising awareness of new treatments as they arise, understanding the neuropsychological and neurocognitive symptoms, prioritizing lost to follow-up patients who are motivated, using new outreach methods, and solidify tracking and identifying methods for patients with PKU [12]. We considered these recommendations in designing our study. The PKU Lost to Follow-Up Recommendations Group found that the greatest factor motivating these patients to return to clinic was the availability of a new pharmacologic treatment. The new treatments currently clinically available as well as many in clinical trials made this an opportune time to re-connect with our lost to clinic population. In 2020, the PKU Lost to Follow-Up Recommendations Group delved into the reasons for adults with PKU not returning to clinic. These earlier findings included the following main reasons: “discharge from the clinic as a child”, “personal choice”, “strict dietary and treatment guidelines”, “treatment costs”, “lack of insurance coverage for medical formula or foods”, “lack of adult-specific medical formula or foods”, “insufficient support systems”, “unavailability of adult PKU clinics”, and “factors inherent to managing a chronic medical condition, including the effort to maintain Phe levels over a sustained period of time” [13].

There was one study published in 2010 about a single center's attempt to find patients lost to care and try to re-engage them [14]. They identified individuals with PKU who had not been in clinic for 2 or more years. Through outreach efforts including letters, mailed questionnaires, phone contact, and advertised PKU educational sessions, they successfully re-established connection with 63/162 eligible patients, with 21/63 returning to treatment. They noted factors contributing to lack of follow-up including inadequate health insurance and challenges to dietary treatment.

Over the last five years, our team of providers in the Dr. Harvey Levy Program for PKU and Related Conditions at Boston Children's Hospital has cared for 318 individuals with PAH deficiency. Our clinic had a recent quality improvement project to reach patients out of clinic between 2 and 5 years (unpublished). We have identified an unmet need for education in the adult PKU population through the published literature and our personal experience with patients. Some individuals with PKU had returned to our clinic after many years or decades, having learned themselves about changes in guidelines. We have experienced patients who were dismayed that they were never informed about changes in management guidelines or novel pharmacologic treatments. This is the impetus of this project, to reinitiate contact with previous patients who have been lost to care.

The primary objective of this study was to contact patients formerly followed at the Boston Children's Hospital PKU Clinic without follow-up for 5–50+ years with the aim of informing them about changes in treatment guidelines and the types of treatments currently available. Our goal was to offer the possibility of returning to clinic for care, monitoring, and treatment. The secondary objectives were to gather patient-volunteered information on why they did not return to clinic and on their self-reported health and wellbeing during their years without follow-up. Our final goal, regardless of whether or not individuals returned to clinic, was to capture contact information and consent to re-contact in order to maintain periodic communication; in this way we sought to create a bridge between our clinic and this population for longitudinal follow-up and for sharing future advancements in PKU over time. Here, we present our findings and experience in educating patients, returning patients to clinic, and understanding why patients become lost to clinical care.

## 2 Methods

We obtained IRB exempt status and completed discussions with our hospital compliance officers about approved modalities for searching and contacting former patients. We aimed to contact individuals who had been previously treated or monitored for PAH-deficiency requiring treatment in the Boston Children's Hospital PKU Clinic, and who had no clinic visits for five or more years, with the aim of having a zoom-based educational session and structured interview (“study visit”) following this initial outreach. We excluded from eligibility individuals with mild PKU or hyperphenylalaninemia (HPA) not requiring treatment (levels not exceeding 360 μmol/L), with the exception of women of child-bearing age who would require Phe monitoring in the event of a pregnancy. We excluded individuals with intellectual disability (ID) who would be unable to consent for study participation.

Individuals with a diagnosis of HPA or PKU who used to follow at Boston Children's Hospital were identified through electronic or paper medical records and NBS records. We searched the electronic medical record (EMR) by diagnosis code, PKU (E70.0). Other hospital records used included hospital-archived paper patient charts, dietitians' spreadsheets of patients' Phe levels, genotyping records, and other patient lists from providers, especially from those providers who had practiced at our center the longest.

A list of previous positive PKU NBS test referrals to Boston Children's Hospital was provided by the Massachusetts NBS program, which had records electronically since approximately the year 2000. We also worked with a regional PKU organization, the New England Connection for PKU and Allied Disorders (NECPAD), to send an email blast to members.

The last visit dates were collected from electronic or paper records and used to establish the number of years since last clinic visit. For three individuals, precise last visit dates from over 40 years prior were unobtainable from historical records. These individuals were successfully contacted and able to provide their approximate ages at their last clinic visits, which was used to establish the estimated number of years since their last clinic visits.

The database of patients was created using RedCap, which was also used to document contact attempts and information about each individual. Using the last known contact information for the patient, telephone contact was attempted, and then email and/or mail. In efforts to further protect patient privacy, web-based searches for updated telephone numbers were conducted in privacy mode on search engines, which disables tracking and data storing from the web browser. Once a valid phone number was identified, multiple attempts were made to reach the patient.

Significant efforts were undertaken during the initial contact phase to protect the patient's privacy. Prior to confirmation of the patient's identity, only the patient's first name was used; the diagnosis of PKU was not mentioned nor were specifics of the clinic. A vague explanation was initially offered, with study team callers explaining that Boston Children's Hospital was attempting to contact former patients. Individuals were asked to confirm their identity using their full name and date of birth before any other specifics about the individual or about the study were discussed.

When successful contact was made and identity was confirmed, a scheduled phone call or Zoom-based meeting with a metabolic physician and dietitian was offered as the study visit. The main objective of the study visit was to serve as an educational intervention. During the study visit, the patient was also asked questions in a structured interview about their historical and current PKU management (if any), previous engagement with PKU clinic, reasons for not returning to clinic, as well as goals and hopes for PKU management. Participants were asked to self-rank their quality of life, physical health, and mental health each on a scale from 1 to 10 with 10 being the highest [Supplement 1]. The physician and dietitian then provided information with discussion and visual aids regarding current treatment guidelines and new treatment developments, including medication and diet management strategies, highlighting what was new since the patient last received PKU care. For those interested, information on emerging therapies and research in PKU management was also provided. At the end of the study visit, individuals who expressed interest in returning to clinic received assistance with scheduling clinic visits.

When individuals were successfully contacted and with their consent, we stored their accurate contact information in our clinic records, with the plan of future engagement with those who had expressed interest in periodic communication from the Boston Children's Hospital PKU Clinic.

## 3 Results

**Fig. 1:**
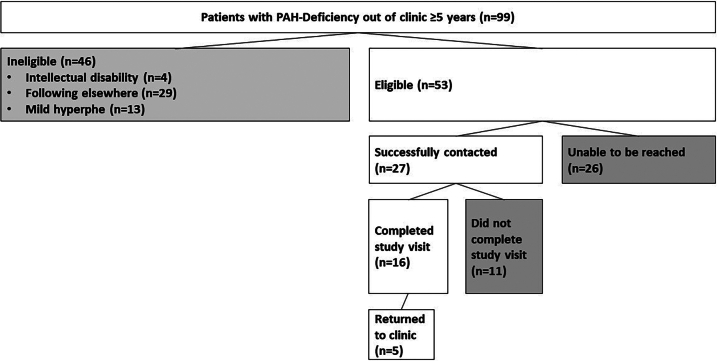
Outcomes of patient population identified by PKU clinic outreach effort.

Ninety-nine former patients with PAH-deficiency who had not been to clinic in ≥5 years were screened for eligibility [Fig. 1]. There were 46 ineligible because they either had mild HPA not requiring treatment, had documented intellectual disability, or were not truly lost to care. The last group either had documented relocation and/or referral to another center or we confirmed through our outreach that they were following at another clinic. Out of 53 eligible, 29 were female and 24 male, with ages ranging from 26 to 67 years (4 in their 60s, 17 in 50s, 10 in 40s, 13 in 30s, 9 in 20s). The median age at the last clinic visit date was 35.7 years for females and 27.1 years for males, suggesting that females with PKU followed in this clinic longer or else returned to clinic for family planning. There was a documented pregnancy visit for 15/29 females. The time away from clinic ranged from 5 to 45 years (median 12.4 years, with 3 individuals with precise last visit dates unknown and estimated times away from clinic based on their individual memory) [Table 1]. After multiple attempts to contact these 53 individuals, 27 were successfully contacted. Eleven out of the 27 successfully contacted never moved forward with the study, including 4 who did not show up for multiple scheduled study visits, and 7 who were not interested in scheduling a study visit. Sixteen out of 27 completed the study visit, and 5 of those 16 have returned to clinic [Fig. 1]. Since returning to clinic following this outreach effort, one individual started on pegvaliase, another expressed interest in pegvaliase, and another expressed interest in sapropterin.

**Table 1:** Ages and years from clinic for eligible cohort lost to PKU Clinic (*n* = 53).

	**Years since last clinic visit**		**Current age** **(median years)**		**Age at last clinic visit** **(median years)**			**Participants with pregnancy visit(*s*)**^d^
	Median (25%ile, 75%ile)	Range^a^	Median (25%ile, 75%ile)	Range	Total (25%ile, 75%ile)	Male^b^	Female^c^	
Eligible cohort (n = 53)	12.4 (8.4, 17.2)	5–45	46.9 (32.8, 53.3)	26–67	32.3 (21.1, 38.2)	27.6	35.7	15
Study visit cohort (*n* = 16)	12.8 (11.8, 14.8)	7–44	50.3 (38.2, 56.2)	29–60	35.2 (20.9, 42.4)	27.1	37.5	4

a 3/53 eligible patients had unknown last visit dates that predated available medical records

b Total eligible cohort *n* = 24; study visit cohort *n* = 6

c Total eligible cohort *n* = 29; study visit cohort *n* = 10

d Number of patients with one or more documented pregnancy visits

Of the 16 who completed the study visit, 6 were male and 10 were female. The median current age of the participant cohort was 50.3 years. Three were aged in their 60s, 4 in their 50s, 4 in their 40s, 4 in their 30s, and 1 in their 20s. Their average time away from clinic had been 14.7 years (median 12.8 years), and age of the last clinic between males and females also differed, with the median age at last visit for males 27.1 years and females 37.5 years. Four out of ten females had previously returned to clinic for one or more pregnancies [Table 1].

Two patients interviewed had previously received monitoring but never received treatment as their Phe levels had not exceeded the upper limit of the treatment threshold at the time; both would be eligible for monitoring and possible treatment in pregnancy, and one of the two was self-restricting protein intake due to self-reported symptoms with higher protein intake. The remaining 14 had all been on a traditional PKU diet including low protein food intake with PHE-free PKU formula. One of these 14 continued to follow a low protein diet and had been receiving PKU formula with the assistance of a primary care physician (PCP) but without any recent blood Phe level testing and without any follow-up from a dietitian or physician from PKU clinic. The remaining 13/14 individuals who had been on diet in childhood had all liberalized the diet and stopped taking PKU formula by the time they reached the age of 18. Age of diet liberalization and formula discontinuation varied from age 5 to age 18, with 6 individuals having retried or resumed the diet at some point after initial discontinuation, four of whom did so for pregnancies. Despite coming off diet, three individuals continued to avoid or restrict meat and other high protein foods to varying degrees. As for additional treatment, three out of 14 had been on sapropterin, with 2/3 having stopped due to insurance difficulties and 1/3 who had discontinued due to having no response to the medication.

Two out of the 16 were not working (one by choice and one with a recent job loss) and the remaining 14 were working either full or part-time. Nine were working in jobs with a professional classification, requiring post-high school education or certification. Level of education varied up to doctorate level.

Using a 1 to 10 rating scale with 10 as the best, the average self-reported ranking in physical health was 7.9 (range 5–10), in mental health 8.0 (range 3–10), and in quality of life 8.2 (range 2–10). One individual self-rated the lowest scores in all three categories noting significant concerns about a non-PKU chronic medical condition, as well as depression and anxiety.

Twelve individuals were married or in a long-term partnership and four were single. Eight individuals had children, including five females, four of whom who had previously returned to clinic for pregnancies and one with a child by adoption.

The most commonly shared reasons for not returning to clinic were discharge from clinic as a child (7/16) and insurance coverage issues (6/16); other reasons included financial or time constraints, lack of support from clinic providers or family members, and feelings of disconnectedness with the PKU community or with treatment recommendations at the time. Individuals shared feelings that diet treatment had not worked well for them; one individual shared strong negative feelings having endured physical punishment by parents when Phe levels were elevated as a child. Others expressed not experiencing any negative impacts after coming off diet in childhood, sharing their successes with school, career, and high quality of life. Others expressed difficulties with headaches and difficulties with school and work, possibly attributable to untreated PKU.

Symptoms associated with untreated PKU were noted in 11/16 individuals interviewed, either documented during previous medical visits or volunteered during their study visit. These symptoms included anxiety, depression, learning difficulties, challenges with focusing, organization or time management, and the need for additional time for certain tasks.

When asked about their hopes and goals for PKU treatments, participants were interested in convenient treatments that would align with their overall health goals. Overwhelmingly 15/16 participants were interested in returning to clinic now or in the future with only one participant expressing that they were not interested. All participants wanted to be contacted about future research and treatment.

## 4 Discussion

This study aimed to address our clinic's PKU patient population identified by NBS who have become lost to care. In the early years of NBS for PKU, which began in 1962, children were generally treated for part of childhood with a Phe-restricted diet plus the addition of Phe-reduced PKU formula with the goals of controlling blood Phe levels and still providing total nutrient adequacy. A publication in 1971 stated that dietary restriction to prevent intellectual disability was unnecessary beyond 6 years [15]. In 2000, the NIH consensus statement issued a recommendation for “diet for life;” however, those previously discharged from PKU programs across the country were often not aware that this guidance had changed.

In our center's experience, those in the early years of NBS were treated until age 6–14, with variation based on shared decision making between the physician and family. This accepted practice of the time resulted in an older generation of adults with PKU who were mostly taken off diet and discharged from PKU clinic during childhood or adolescence. Some individuals in their 20s and 30s who were advised from early childhood to remain on diet for life were unable to sustain this form of treatment and therefore self-discontinued care. Many remained separated from clinic; however, over the past decade, some patients re-engaged in clinic with the initiation of pharmacologic treatment options, such as pegvaliase and sapropterin. Others re-engaged for clinical trial participation including medication and gene therapy trials.

We emphasize that our colleagues treating the earliest population of patients identified by NBS were acting on the best information available at that time. We also recognize that these former practices of discharging children and adolescents from care have contributed to the current PKU adult population having been under-treated. When the guidelines changed to recommend treatment for life in 2000, our center had put forth an effort to reach former patients to inform them of the change, and one individual we spoke with recalled receiving a letter with this update. We do not know the extent of this effort, but contacting 37 years of patients in the early years of the internet would have been a formidable task. Clearly, from our center's experience, and that of other PKU centers, adults with PKU are an under-served population. In consideration of this healthcare inequity, we may consider the extent of our obligation to reach former patients who may not be aware of dramatically changed guidelines and new treatments.

**Fig. 2:**
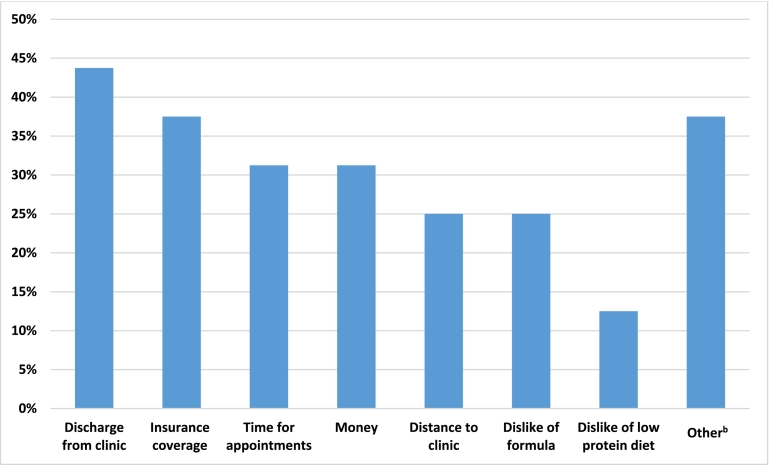
Reasons for not returning to clinic based on LTFU patient responses in MD/RD interviews^a^. ^a^16 total individuals interviewed, each with option to select multiple reasons for not returning to clinic. ^b^Other reasons for not returning to care documented from interviews and indicated in manuscript.

Our study found that discharge from clinic was the most common reason for being lost to care in our cohort [Fig. 2]. Some individuals reported that they had maintained clinic contact for some time after their discharge, but they had lost connection to providers over time. From our individual discussions, those born in the 1960's mostly had not returned to clinic due to lack of knowledge that it might be of interest to return to care. Some individuals we spoke with were startled to discover that their blood Phe levels were 10–20 times the upper limit of normal, and that this could be impacting their functioning in adulthood. The next most common reason for not seeking PKU treatment was inadequate insurance coverage (6/16), and then time and money (each 5/16). The least cited reasons for not seeking PKU treatment were distance to clinic (4/16) and dislike of the PKU dietary regimen including dislike of formula (4/16) and dislike of the low protein diet (2/16) (Fig. 2). Six individuals cited “Other” reasons including lack of support from clinic providers or family, feelings of hopelessness about treatment options, the mental and emotional toll of seeking treatment as an adult within a children's hospital, and difficulties with scheduling and prioritizing appointments.

Our study attempted to reach patients who had been lost to our clinic for 5 to 50+ years. This made our study different from the Burton 2010 lost to care study and our own center's previous initiative to re-engage patients out of clinic for 2 or more years. In our experience, many patients out of clinic for 2 years are still on treatment and/or consider themselves to be active clinic patients. The ambitious goal of this study was to reconnect with historical patients out of care for a much longer time period, as by 5 years they are disassociated with the clinic; therefore, it was anticipated to have a lower rate of contact and return to clinic than the above efforts.

We identified 53 patients who had been lost to care for a mean of 16 years (median of 12.4 years). The oldest individual diagnosed by Massachusetts NBS would be 61 years old at the time of this manuscript. We expected fewer young adults to be lost to care compared to middle aged adults; however, we identified nearly as many younger adults, likely because they had been followed in our clinic in the era of the EMR and were therefore easier to identify. Based on percentage of successful contacts by decade, these younger adults did not prove easier to connect with. For some it may have been their preference not to continue following with the PKU clinic.

Within the eligible cohort of this study, females continued to engage with clinic for almost eight years longer than males, with 15 having had documented pregnancy-related clinic visits. Women and their providers presumably understood that continuation of PKU care and monitoring was vital into child-bearing years in order to avoid maternal PKU effects, and providers may have been more diligent in reinforcing females' continuation of care with this in mind. At least one woman cited PKU as a reason for not wanting to have children.

Of those who completed the study visit, most were employed, over half in professional positions. Most were married or in a long-term partnership. Half (8/16) had children, and some participants were of reproductive age and may have children in the future. Physical health, mental health and quality of life were all self-ranked to be on average around 8/10, with 10 being the highest. Despite the high self-ranking, many volunteered experiencing symptoms that could be attributable to untreated or undertreated PKU including anxiety, depression, learning difficulties, challenges with focusing, organization or time management, and the need for additional time for certain tasks.

A minority of the individuals we spoke to had tried sapropterin and none had taken pegvaliase. One individual was actively following a low-protein diet with PKU formula but was not followed by a PKU program. Another 3/16 were restricting protein intake to some extent. Most participants had knowledge gaps about newer treatments for PKU including nutritional products and medications. Most expressed interest in returning to clinic after the educational component of the meeting, and all desired future contact regarding research and treatment opportunities. We noted a desire for connectedness with clinic and the PKU community even though they had been lost to care for large amounts of time.

We acknowledge many limitations with this study, largely due to the difficult feat of identifying individuals with PKU who were diagnosed and discharged many decades ago. We identified individuals who had not returned to clinic through all available means including paper chart review, provider interviews, EMR, paper and digital treatment and research records kept by clinic providers, and NBS program records. Yet, we had limitations as the NBS program could only provide records after the year 2000 and our hospital's EMR was only available since around 1995. Contact information from paper chart and EMR were mostly outdated and may have lacked changes in surnames. Our hospital was using a Cerner-based EMR, without a broad link to other hospitals, which may have limited our retrieval of more up-to-date contact information. Our additional search methods were all electronic, and finding individuals was therefore contingent on public availability of their contact information.

There were additional IRB-imposed restrictions aimed at avoiding dissemination of protected health information. Some methods of addressing these limitations included the use of privacy protected web browsers. We also sent physical mailings with confidentiality warnings. When attempting contact by phone, we needed to first verify an individual's identity, which required that the person share their birth date and full name prior to our providing any specific information about the reason for the call or the clinic we were calling from. This stringent privacy requirement may have contributed to loss of participants. We also noted a distrust in the medical community from some individuals we contacted. During phone calls it was therefore helpful to mention previous providers to gain trust and credibility.

Some were not available during typical work hours, and others unable or unwilling to pick up or return calls. We implemented evening calls after general working-hours. However, we found that even once we reached individuals, many did not show-up for scheduled study visits, despite many attempts by the study team to cater the time and method to suit the individual.

Having completed our attempt at contacting 60 years of patients identified by NBS and not currently in clinic, we are exploring ideas for sustaining our efforts. Using this data and the experience from this study, we would like to develop a process internally to prevent patients from becoming lost to care. For those actively managed in clinic, strategies may include timely post-clinic telephone or telemedicine check-ins following changes or initiation of therapy as well as a system to flag patients due for routine follow-up visits. Telemedicine can remove many barriers including time, distance, child care, and money involved in coming to clinic in person, particularly from a distance. We have maintained a database with current and past patients, and plan to update yearly with visit dates to find anyone needing clinic follow-up. We will develop a plan for periodic re-contact for those not being seen in clinic in order to offer updates on new treatment or research opportunities or news in the PKU community. We have also encouraged connecting with the regional and national PKU organizations. Through this study, we have identified a group who have some concerns about how PKU will affect them long-term, but the majority have no desire to treat with diet or medications.

We noted significant variation in individuals' past experiences and current outlooks on PKU treatment, and were touched by stories about deep connections to historic PKU providers as well as influential caregiver and family dynamics. While we acknowledge the ascertainment bias of this study given the participants in the educational intervention were willing to engage with our center through the study visit, we were nevertheless struck by how adults who chose not to return to care wanted to remain informed and were interested in being re-contacted. All individuals interviewed indicated that they would like to be re-contacted periodically with treatment updates and for connection to the PKU clinic and community.

## Author disclosure

This project was funded as a sub-award through the PHEFree Consortium.

Stephanie Sacharow, MD has has been a research investigator for or has participated in advisory boards and/or educational programs for Biomarin, Synlogic, Jnana, PTC Therapeutics, Inc.

Suzanne Hollander, MS, RD, LDN has participated in funded research or consulting/advisory boards for Biomarin, PTC Therapeutics, Inc., Cycle Therapeutics, Sanofi Genzyme, and Vitaflo.

## CRediT authorship contribution statement

**S. Sacharow:** Writing – review & editing, Writing – original draft, Supervision, Resources, Project administration, Methodology, Investigation, Funding acquisition, Data curation, Conceptualization. **E. Zhu:** Project administration, Investigation. **S. Hollander:** Writing – review & editing, Writing – original draft, Supervision, Project administration, Methodology, Investigation, Formal analysis, Data curation.

## Declaration of competing interest

None

## Data Availability

The data that has been used is confidential.

## Appendix A Supplementary data

Supplementary material Image 1
