# Supplementary material for: The challenge of adults with phenylketonuria who have been lost to care; a single center's attempt to reach those diagnosed with PKU over 60 years of newborn screening
Source: Mol Genet Metab Rep. 2024 Jun 8;40:101099. doi: 10.1016/j.ymgmr.2024.101099 (PMC11219965; doi:10.1016/j.ymgmr.2024.101099)
Supplement: Supplementary file 1 — Supplementary material [file mmc1.docx]

Figure 1. Outcomes of patient population identified by PKU clinic outreach effort
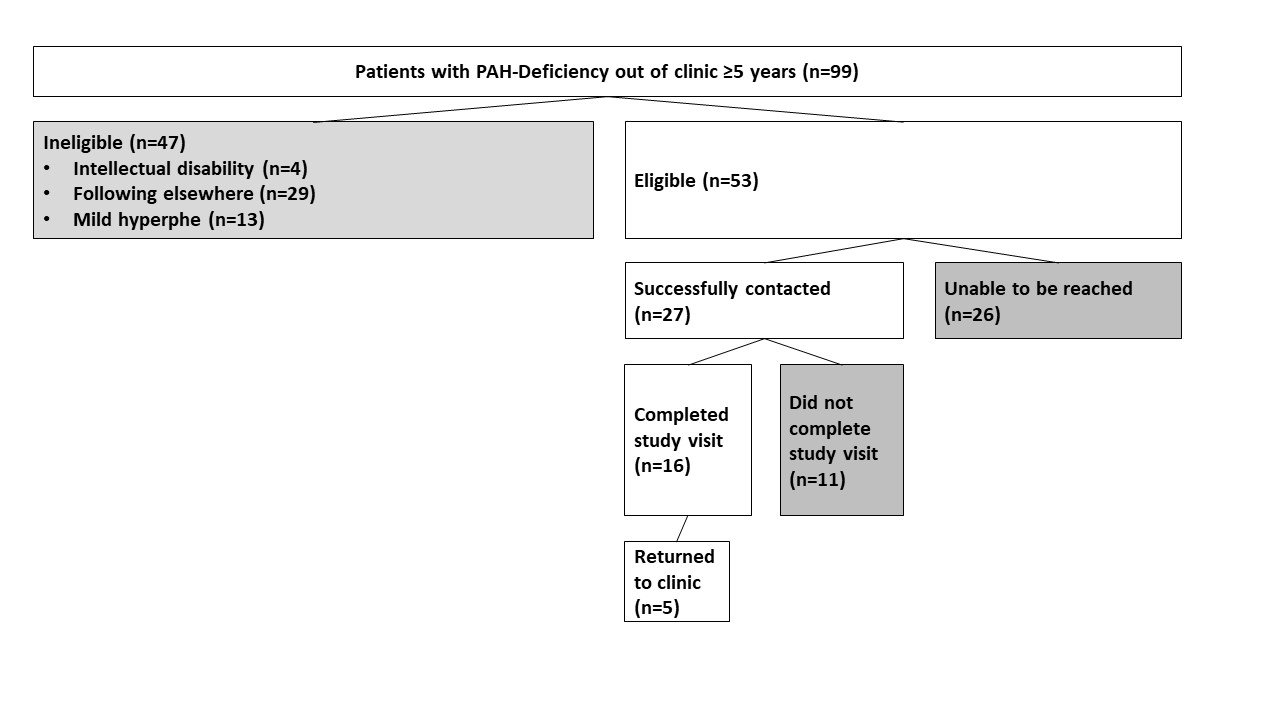


Supplement 1. Structured interview questions during study visit

**Personal background information**

- Are you working? [Yes/No]
  - What type of work do you do? (*Details recorded if provided)*
- Are you in a relationship? [married, single, divorced, widowed, other partnership] (*Details recorded if provided)*
- Do you have children? [Yes/No] (*Details recorded if provided)*

**Timing of last engagement with clinic & past/current treatment**

- When was the last time you were followed by a PKU program?
- Why did you stop following in PKU clinic? [multiple selections: Discharge from clinic as a child, Distance to clinic, Time for appointments, Money (copays, special foods), Insurance coverage (for visits, formula), Do not like drinking formula, Do not like eating a low protein diet, Other *(details recorded)*]
- Are you currently on treatment for PKU? What type of treatment? [Suggested wording: *“We know that more than half of adult with PKU are not currently following with a clinic and are not on treatment. Are you currently doing any sort of treatment for PKU?”]*
- What was your previous PKU treatment? [multiple selections: diet, sapropterin, other]
- Rate physical health [1-10 scale, 10 highest]
- Rate mental health [1-10 scale, 10 highest]
- Rate quality of life [1-10 scale, 10 highest]
  - *Details of physical health, mental health, and quality of life recorded when provided*

**Interest in contact and clinic**

- Do you have any interest in returning to PKU clinic now or in the future?
  - In-person or telemedicine?
  - Doctor only? Or Doctor plus dietitian?
- Can our clinic contact you about future treatments or PKU research opportunities?

Table 1. Ages and years from clinic for eligible cohort lost to PKU Clinic (n=53)

|  | **Years since last clinic visit** | | **Current age (median years)** | | **Age at last clinic visit  (median years)** | | | **Participants with pregnancy visit(s)^d^** |
| --- | --- | --- | --- | --- | --- | --- | --- | --- |
|  | Median (25%ile, 75%ile) | Range^a^ | Median  (25%ile, 75%ile) | Range | Total  (25%ile, 75%ile) | Male^b^ | Female^c^ |  |
| Eligible  cohort (n=53) | 12.4  (8.4, 17.2) | 5-45 | 46.9  (32.8, 53.3) | 26-67 | 32.3  (21.1, 38.2) | 27.6 | 35.7 | 15 |
| Study visit  cohort (n=16) | 12.8  (11.8, 14.8) | 7-44 | 50.3  (38.2, 56.2) | 29-60 | 35.2  (20.9, 42.4) | 27.1 | 37.5 | 4 |
| ^a^3/53 eligible patients had unknown last visit dates that predated available medical records ^b^Total eligible cohort n=24; study visit cohort n=6 ^c^Total eligible cohort n=29; study visit cohort n=10  ^d^Number of patients with one or more documented pregnancy visits | | | | | | | | |

Figure 2. Reasons for not returning to clinic based on LTFU patient responses in MD/RD interviews^a^

**Other^b^**

^a^16 total individuals interviewed, each with option to select multiple reasons for not returning to clinic

^b^Other reasons for not returning to care documented from interviews and indicated in manuscript
